# Radiation pressure measurement using a macroscopic oscillator in an ambient environment

**DOI:** 10.1038/s41598-020-77295-5

**Published:** 2020-11-24

**Authors:** Mikko Partanen, Hyeonwoo Lee, Kyunghwan Oh

**Affiliations:** 1 Photonic Device Physics Laboratory, Department of Physics, Yonsei University, 50 Yonsei-ro, Seodaemun-gu, Seoul, 03722 South Korea; 2 Photonics Group, Department of Electronics and Nanoengineering, Aalto University, P.O. Box 15500, 00076 Aalto, Finland

**Keywords:** Optics and photonics, Optical physics, Physics, Optical physics

## Abstract

In contrast to current efforts to quantify the radiation pressure of light using nano-micromechanical resonators in cryogenic conditions, we proposed and experimentally demonstrated the radiation pressure measurement in ambient conditions by utilizing a macroscopic mechanical longitudinal oscillator with an effective mass of the order of 20 g. The light pressure on a mirror attached to the oscillator was recorded in a Michelson interferometer and results showed, within the experimental accuracy of 3.9%, a good agreement with the harmonic oscillator model without free parameters.

## Introduction

According to Newton’s second law, the force ${\mathbf {F}}$ on an object is well known to be equal to the rate of change of the momentum ${\mathbf {p}}$ of the object as ${\mathbf {F}}=d{\mathbf {p}}/dt$. From this fundamental law, we can expect the largest conversion of the optical momentum to the mechanical momentum of a medium when light is fully reflected from a mirror. The magnitude of this force on an ideal 100% reflecting mirror in a vacuum is given by

1 $$\begin{aligned} F=\frac{2P}{c}, \end{aligned}$$

where *P* is the optical power and *c* is the speed of light. If light is irradiated on a non-planar or partly absorbing object, the value of the radiation pressure is always smaller than its maximum value given in Eq. (1). Thus, the measurement of the maximum value of the optical force-power ratio, $F/P=2/c$, which has a universal value given in terms of the speed of light, provides a good test for the accuracy of the radiation pressure measurements.

The radiation pressure of light was first theoretically described by Maxwell^1^ in 1873, and then experimentally measured independently by Lebedev^2^ and by Nichols and Hull^3^ in 1901, but the accuracy of these early experiments was very limited. Despite being a century-old discovery, the radiation pressure continues to be one of the key research interests in current optomechanics, such as in cooling of mechanical resonators^4–8^, solar sail development^9^, ultra-high laser power measurements^10,11^, and nano-scale cantilevers’ spring constant calibration^12,13^, to name a few. Recently, there also has been renewed interest in the centennial Abraham–Minkowski controversy on the light momentum in a dielectric medium^14–24^.

The main trend in light pressure studies in recent years has been to miniaturize a mechanical oscillator to the nano-micro scale for a higher sensitivity to the radiation pressure^4,6,7,25^. However, optical forces in those nano-micromechanical systems have been directly accompanied by photothermal effects due to short thermal time constants of the miniaturized resonators^6,7,26–29^, which has required further sophisticated techniques to discern them from the radiation pressure effects. Therefore, various optical, mechanical, and thermal techniques have been developed to overcome the trade-off between the radiation pressure and the photothermal effects, such as complex resonator designs consisting of highly reflective multilayer coatings deposited on the cantilever to further increase the reflectivity^7,12^, attachment of an additional mass to increase the thermal time constant of the cantilever^13^, or other ways to separate the optical force from the photothermal effects^30,31^.

**Figure 1:**
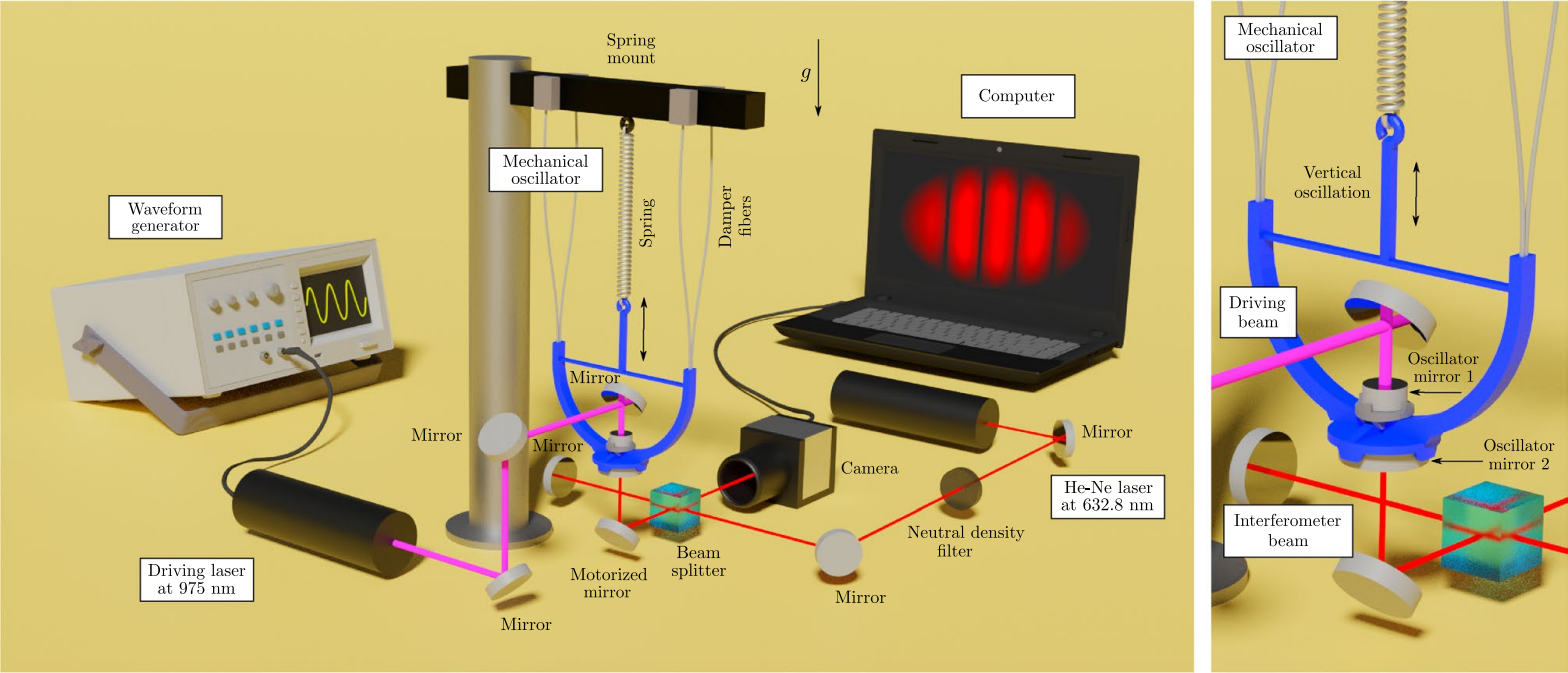
The experimental setup consists of three main parts shown in the left panel: the mechanical oscillator, the diode laser driving the oscillator, and the Michelson interferometer. The right panel focuses on the vertically hanging mechanical oscillator. The mechanical oscillator is driven by a modulated laser beam at 975 nm through the reflection from the highly reflective oscillator mirror 1. The nanoscale oscillation is detected through the oscillator mirror 2 by the Michelson interferometer using the He–Ne laser at 632.8 nm. The motorized mirror below the oscillator is used for the remote tuning of the interference fringe spacing, but it is not actively controlled during the measurements. The illustration includes the damper fibers that are used for increasing the damping constant of the higher damping oscillator. The apparatuses are mounted on an actively damped optical table for isolating the setup from external acoustic and seismic vibrations. The oscillator part of the setup is also protected with plastic walls not shown in this illustration. A more complete description of the experiment is presented in the “Methods” section. (Image created by using Blender 2.8, https://www.blender.org/, and Inkscape 0.92, https://inkscape.org/).

In this work, we attempted a new direction opposite to the current trends by achieving a quantitative measurement of the radiation pressure of light in an ambient environment at room temperature by utilizing a macroscopic mechanical harmonic oscillator, which is orders of magnitude heavier than oscillators in previous reports^30–35^. The experimental setup is illustrated in Fig. 1. In contrast to conventional torsional oscillators used in most of the previous measurements, our oscillator is designed to allow only the longitudinal motion. Here we varied the mass and the damping constant to verify the accuracy of the harmonic oscillator model in the radiation pressure measurements. Note that our method can obviate the elaborated process to calibrate the spring constants^30,31^, as the only additional measurement of the oscillator parameters is the direct determination of the oscillator masses using a digital scale.

## Mechanical oscillator

The oscillator is driven optically by the reflection of the modulated laser beam at the wavelength of 975 nm at a highly reflective dielectric mirror, which is the oscillator mirror 1 in Fig. 1. The laser diode at this wavelength has been widely used to pump optical amplifiers and provides a good power stability of $\sim \pm 0.5\%$. The effective total reflectivity of the oscillator mirror 1 was larger than 99.9% (see “Methods”) and Eq. (1) can be used to quantify the optical force. The longitudinal displacement of the oscillator was detected by a Michelson interferometer using another laser at the wavelength of 632.8 nm. The interferometer beam was reflected from the oscillator mirror 2. The shifts of the interference fringes were recorded using a camera at a frame rate of 200 frames per second, from which the displacement of the oscillator was estimated for various incident light powers. It is noteworthy, in particular, that photothermal effects can be excluded since light is reflected from a highly reflective dielectric mirror on a macroscopic mechanical oscillator whose thermal time constant is much longer than the modulation time of the laser field. A more complete description of the experimental setup is presented in the “Methods” section.

Newton’s equation of motion for the mechanical oscillator with an effective mass *m* is given by^4^

2 $$\begin{aligned} \frac{d^2x}{dt^2}+2\zeta \omega _0\frac{dx}{dt}+\omega _0^2x=\frac{F}{m}, \end{aligned}$$

where $\omega _0$ is the undamped resonance frequency of the harmonic oscillator, $\zeta$ is the damping coefficient, and *F* is the net external force. The damping coefficient is related to the Q factor as $Q=1/(2\zeta )$. When the mass of the vertically aligned spring is not negligible, the effective mass of the oscillator is given by $m=m_0+m_{{\mathrm {s}}}/3$, where $m_0$ is the rest mass of the oscillator and $m_{{\mathrm {s}}}$ is the rest mass of the spring^36^.

If the force is harmonically modulated with the angular frequency $\omega$ as $F=F_0\cos ^2(\frac{1}{2}\omega t)=\frac{1}{2}F_0[1+\cos (\omega t)]$, where $F_0$ is the peak to peak amplitude of the force, then the steady-state solution of Eq. (2) is given as $x(t)=x(\omega )\cos (\omega t+\varphi )+F_0/(2m\omega _0^2)$, where $\varphi =\arctan [2\omega \omega _0\zeta /(\omega ^2-\omega _0^2)]\in [-\pi ,0]$ and the displacement amplitude $x(\omega )$ is given by

3 $$\begin{aligned} x(\omega )=\frac{F_0/m}{2\sqrt{ (2\omega \omega _0\zeta )^2+(\omega ^2-\omega _0^2)^2}}. \end{aligned}$$

The resonance frequency of the significantly underdamped oscillator with $\zeta <1/\sqrt{2}$ is $\omega _{{\mathrm {r}}}=\omega _0\sqrt{1-2\zeta ^2}$^36^. At this frequency, the displacement amplitude of the oscillator in Eq. (3) obtains its peak value, given by $x_0=F_0/(4m\omega _0^2\zeta \sqrt{1-\zeta ^2})$. Thus, from the measured peak value of the displacement amplitude, we can obtain the optical force as

4 $$\begin{aligned} F_0=4m\omega _0^2\zeta \sqrt{1-\zeta ^2}\,x_0. \end{aligned}$$

Here, for our macroscopic oscillator, the undamped angular frequency and the damping constant can be accurately determined based on the position and width of the mechanical resonance peak and the effective mass of the oscillator can be determined from the oscillator and spring masses measured with a digital scale. The effective mass of the lower damping oscillator without the damper fibers in Fig. 1 is $m=(18.363\pm 0.001)$ g, while the effective mass the higher damping oscillator with the damper fibers is $m=(19.007\pm 0.001)$ g. Note that the difference in the oscillator masses is mainly produced in their fabrication and it is not due to the damper fibers whose total mass is less than 0.2 g. The damper fiber is commercially available optical fiber (Thorlabs, FG105LCA), which provides a very high mechanical stability against tensile stress.

**Figure 2:**
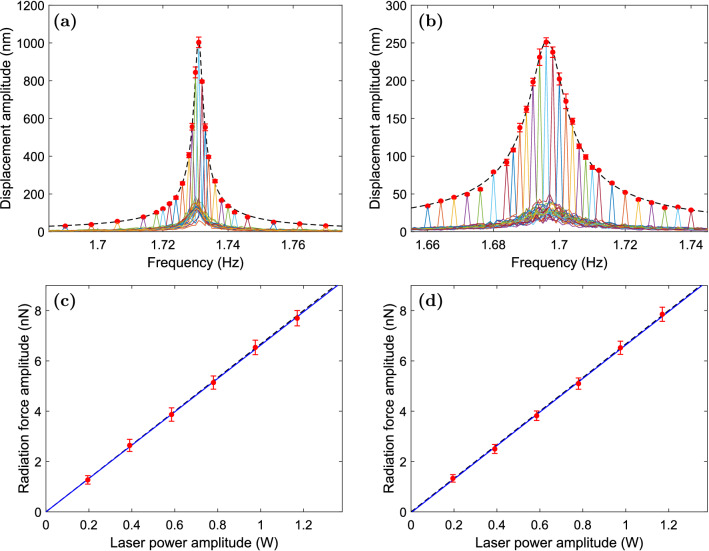
The measured displacement amplitude of the mechanical oscillator is plotted (**a**) for the lower damping oscillator and (**b**) for the higher damping oscillator as a function of the modulation frequency of the driving laser with an example peak to peak power amplitude of $P_0=0.975$ W. Each graph that is marked with a solid line is the averaged frequency spectrum of a measurement for a single modulation frequency. The graphs peak at the modulation frequency and the peak points are marked with red dots. The modulation frequency is varied around the resonance frequency of the mechanical harmonic oscillator. The peak points of the graphs form together a curve that is the response function of the mechanical harmonic oscillator. The fitted harmonic oscillator response function is marked with the dashed line. In (**c**) and (**d**), the measured peak to peak radiation force amplitude is plotted for the two oscillators as a function of the peak to peak laser power amplitude. The least-squares regression lines are marked with the solid lines. The linear theoretical curve $F_0=2P_0/c$ is presented by the dashed lines. The horizontal error bars corresponding to the $\pm \,0.5\%$ uncertainty of the laser power are not shown because of their smallness.

## Results

Figure 2 presents the experimental results. In Fig. 2a, the measured displacement amplitude of the lower damping oscillator is plotted as a function of the modulation frequency of the driving laser field with an example peak to peak power amplitude of $P_0=0.975$ W. Fig. 2b presents the same plot for the higher damping oscillator. Each graph that is marked with a solid line is the averaged frequency spectrum of a measurement for a single modulation frequency. The measurement time is an integer multiple of the modulation period close to 1000 s and the ensemble averaging is made over 10 or more measurements. The error in the displacement amplitude is the standard deviation of the average and it could be made smaller by averaging over a larger number of measurements.

In Fig. 2a,b, one can see that the fitted harmonic oscillator response function in Eq. (3) accurately describes the experimental results of both oscillators. It can be noted that, in the presence of photothermal effects, this response function would be modified from the ideal harmonic oscillator form as described, e.g., in Refs.^30,31^. Thus, the ideal harmonic oscillator form of the frequency response function in Fig. 2a indicates that photothermal effects are negligible in our macroscopic setting as expected. In Fig. 2a,b, one can also see that the mechanical resonance peak is observable in the noise spectrum that is seen below the fitted harmonic oscillator response function.

**Table 1:** Comparison of the orders of magnitudes of characteristic physical quantities, i.e., mechanical frequency $f_0$, effective oscillator mass *m*, laser power modulation amplitude $P_0$, peak displacement amplitude $x_0$, force amplitude $F_0$, and quality factor *Q*, in the present and selected previous works on the measurement of optical forces with mechanical oscillators. For direct comparability, the quantities in the table are obtained by fitting the harmonic oscillator response function of Eq. (3) to the experimental data corresponding to Fig. 2a or Fig. 2b of the present work. In the column for the optical force per power, $F_0/P_0$, the uncertainties do not account for the uncertainties in the determination of the optical power, which is separately shown in the last column.

Work	$f_0$ (Hz)	*m* (kg)	$P_0$ (W)	$x_0$ (m)	$F_0$ (N)	*Q*	$F_0/P_0$ (1/*c*)	$\Delta P_0/P_0$ (%)
Present	1	$10^{-2}$	1	$10^{-7}$	$10^{-8}$	$10^2$	$1.998\pm 0.077^{{\mathrm {a}}}$	0.5
Weld et al. 2006^13^	$10^2$	$10^{-9}$	$10^{-6}$	$10^{-9}$	$10^{-14}$	$10^4$		
Wilkinson et al. 2013^12^	$10^2$	$10^{-7}$	$10^{-3}$	$10^{-9}$	$10^{-11}$	$10^3$	$1.974\pm 0.116^{{\mathrm {b}}}$	1
Wagner et al. 2018^33^	$10^3$	$10^{-8}$	$10^{-4}$	$10^{-11}$	$10^{-10}$	$10^3$	$1.656\pm 0.043^{{\mathrm {c}}}$	8
Melcher et al. 2014^35^	$10^4$	$10^{-9}$	$10^{-3}$	$10^{-10}$	$10^{-13}$	$10^3$		
Kleckner et al. 2006^7^	$10^4$	$10^{-11}$	$10^{-3}$	$10^{-7}$	$10^{-12}$	$10^5$	$2.113\pm 0.163^{{\mathrm {d}}}$	20
Ma et al. 2018^31^	$10^4$	$10^{-12}$	$10^{-4}$	$10^{-10}$	$10^{-12}$	10	$1.234\pm 0.235^{{\mathrm {e}}}$	3
Ma et al. 2015^30^	$10^4$	$10^{-13}$	$10^{-3}$	$10^{-8}$	$10^{-11}$	10	$0.521\pm 0.010^{{\mathrm {f}}}$	5
Evans et al. 2014^34^	$10^4$	$10^{-14}$	$10^{-5}$	$10^{-10}$	$10^{-13}$	$10^2$		
Gigan et al. 2006^6^	$10^5$	$10^{-11}$	$10^{-3}$	$10^{-10}$	$10^{-11}$	$10^4$		

^a^Determined from the slope of the line in Fig. 2d.

^b^Determined as $F_0/P_0$, where $F_0=kA_0$ with *k* being the spring constant obtained from the nanoindenter and the off-resonance amplitude $A_0=2P_0/(m\omega _0^2c)$ is obtained from the quantities given in Table I of the reference.

^c^Determined from the values in Table 1 of the reference as $F_{{\rm sho}}/P_{{\rm c}}$.

^d^Determined as $F_0/P_0$, where $F_0=kA_{{\rm rad}}$ with given values for the spring constant *k* determined from the thermal vibration spectrum and for the off-resonance amplitude $A_{{\rm rad}}$.

^e^Determined from Fig. 3(b) of the reference at 750 nm. The value is much lower than 2/*c* due to the transmission and absorption which reduce the reflectivity.

^f^Determined from the slope of the line fitted in the experimental data of Fig. 4 of the reference. The value is much lower than 2/*c* due to the partial transmission which reduces the reflectivity.

In the least-squares fitting of the harmonic oscillator response function in the experimental data of the lower damping oscillator in Fig. 2a, the undamped frequency of the mechanical oscillator is found to be $f_0=(1.730943\pm 0.000018)$ Hz and the damping constant is found to be $\zeta =0.000750\pm 0.000012$, which corresponds to the quality factor of $Q=667\pm 11$. The errors indicate the 68.27% confidence intervals, which correspond to one standard deviation of normally distributed quantities. Using the experimental data of the higher damping oscillator in Fig. 2b, we respectively obtain the undamped oscillator frequency of $f_0=(1.696275\pm 0.000073)$ Hz and the damping constant of $\zeta =0.003006\pm 0.000051$, which corresponds to $Q=166.3\pm 2.8$.

Figure 2c shows the measured peak to peak radiation force amplitude of the lower damping oscillator following from Eq. (4) as a function of the peak to peak laser power amplitude. The corresponding radiation force amplitude graph for the higher damping oscillator is presented in Fig. 2d. In both cases, the uncertainty in the laser power is $\pm 0.5\%$. For the lower damping oscillator, the slope of the least-squares regression line is $dF_0/dP_0=(6.63\pm 0.29)\times 10^{-9}\;\text {s/m}=(1.986\pm 0.086)/c$. The relative error is 4.3%, from which 1.6% comes from the determination of the damping constant in the fitting of Fig. 2a and 2.7% comes from the determination of the peak displacement amplitude and the uncertainty of the laser power. These values include uncertainties related to the laser power fluctuations around the expectation value. For the higher damping oscillator, the regression line is $dF_0/dP_0=(6.66\pm 0.26)\times 10^{-9}\;\text {s/m}=(1.998\pm 0.077)/c$, where the relative error is 3.9%, from which 1.7% comes from the determination of the damping constant and 2.2% comes from the determination of the peak displacement amplitude and the uncertainty of the laser power. The slope of the corresponding universal theoretical line, $F_0=2P_0/c$, is $2/c=6.67\times 10^{-9}$ s/m. Thus, the experimental results of both the lower and higher damping oscillators agree with the theory within the experimental accuracy.

That our results are in accurate agreement with the theory is another indication for the insignificance of thermal effects in the present experimental setup. However, the role of thermal effects could be studied in more detail in further experiments. For example, we could follow the closely related experiments by Požar et al.^37^ and repeat the experiment by using mirrors with increasingly higher reflectivities, starting from low reflectivities, in which case thermal effects are surely present, and continuing to ultra high reflectivities, in which case thermal effects become insignificant. We could also accurately verify that the results are independent of the excitation beam radius, as must be the case for true radiation pressure.

Comparison of the orders of magnitudes of the characteristic physical quantities in the present and selected previous works is presented in Table 1. Most notably, it is seen that the effective oscillator mass in the present work is several orders of magnitude larger than the oscillator masses in previous works. Also, the mechanical frequency is lower and the laser power modulation amplitude is larger compared to previous works. The comparison of the optical force per power, $F_0/P_0$, shows that only a few works have used the mechanical oscillator to study the force on an ideal mirror with $F_0/P_0=2/c$ and found an accurate correspondence with this relation as we have done in the present work. Regarding the determination of the absolute radiation force, in contrast to our work, the main experimental uncertainties in most previous works have originated from the determination of the magnitudes of the small optical power and the spring constant of the oscillator.

## Conclusions

In conclusion, we have demonstrated that the radiation pressure of light can be accurately measured in ambient environment by utilizing a macroscopic mechanical oscillator and detecting how the modulation of the optical signal can be tuned to drive the nanoscale motion of the oscillator. We have carried out measurements for two oscillators with different masses and damping constants, and shown that the correspondence between the theory and experiment is obtained within the relative experimental accuracy. The introduced setup can also be used for probing optical forces when the oscillator is driven through the optical fibers that are present as the damper fibers in the setup of Fig. 1, but these investigations related to the Abraham–Minkowski controversy are left as a topic of further work. One might also be able to access additional information on the momentum of light in a medium by immersing the oscillator or its driving mirror in liquids with known refractive indices. However, in the related analysis, one should account for the effects of fluid dynamics and also the surface tension if the immersion is only partial. Our macroscopic oscillator setup and its larger-scale variations can also be used for measuring high laser powers through the determination of the radiation pressure.

## Methods

### Mechanical oscillator

The mechanical oscillator masses are fabricated by 3D printing from polylactic acid, commonly known as PLA. The printing is performed by using the fused deposition modeling (FDM) technique. The design of the oscillator mass includes two mirror mounts and the hook that connects the oscillator mass to the mechanical extension spring, which carries the weight of the oscillator mass. The semicircle form of the oscillator has a mean radius of $R=4.25$ cm. The design of the higher damping oscillator mass also includes the damper fibers (Thorlabs, FG105LCA) whose ends are clued to the oscillator. The rest masses of the lower and higher damping mechanical oscillators are 15.606 g and 16.250 g, respectively. These masses include 1.637 g of the mass of the oscillator mirror 1 (Edmund Optics, 63-129) and 6.715 g of the mass of the oscillator mirror 2 (Thorlabs, BB1-E02).

### Driving laser

The driving laser beam at 975 nm is generated by a multimode laser diode module (Lumics, LU0975T090) and it is directed through a low-loss 0.22 NA silica core multimode fiber (Thorlabs, FG105LCA) to the collimation arrangement after which the laser beam hits the mirror on the mechanical oscillator. The intensity of the driving laser beam is modulated by the waveform generator (Agilent, 33120A) connected to the laser driver (Arroyo Instruments, LaserPak 485-08-05). The temperature of the laser is controlled with the temperature controller (Arroyo Instruments, TECPak 585-04-08). The laser beam coming out from the end of the multimode fiber is collimated by using the achromatic lens (Thorlabs, AC050-008-B) with a diameter of 5 mm and focal length of 7.5mm. After the collimating lens, we use two mirrors (Thorlabs, BB1-E03) for accurately aligning the beam normally to the oscillator mirror 1. In addition, at 80 cm before the mechanical oscillator, we use a focusing lens to adjust the spot size of the laser beam so that the spot diameter is 5 mm when it hits the mirror on the mechanical oscillator. The focusing and diverging angles of the order of one percent are so small that the non-normally incident field components on the mechanical oscillator have negligible influence on our measurement results. Thus, we can safely use the normal incidence assumption in the analysis of the experimental results. We also minimize thermal drifts by using a highly reflective dielectric oscillator mirror 1 with an effective total reflectivity larger than 99.9%. This effective total reflectivity consists of the specular reflectivity $>99.8$% and the estimated normal component of the diffusive reflectivity corresponding to more than half of the light that is not specularly reflected. The reflectivity of this mirror is assumed unity in the analysis, in which case Eq. (1) can be used for the optical force.

### Laser power measurement

The laser power is measured using an optical power meter (Thorlabs, PM400) with an integrating sphere sensor (Thorlabs, S145C) at the position before the laser beam hits the last mirror directing the beam toward the mechanical oscillator. The reduction of the power due to the reflectivity of 99.5% of the last mirror is accounted for in the analysis. The peak to peak amplitude of the sinusoidal modulation was 2.0% smaller than the otherwise stationary beam. Thus, for example, for a stationary beam with a power of 1.000 W, after adding the modulation and accounting for the reflectivity of the last mirror, the peak to peak power hitting the oscillator mirror 1 becomes 0.975 W, which is the value used in the measurements corresponding to Fig. 2a,b.

### Extension springs

In the experiments, we have used three hard extension springs in series to obtain a suitably small total spring constant. The upper and lower springs (Acxess Spring, PE016-312-129000-MW-2500-MH-N-IN) both have a mass of $m_{{\rm s},1}=3.025$ g and a reported extension rate of $k_{{\rm s},1}=5$ N/m, while the middle spring (Acxess Spring, PE016-312-90250-MW-1880-MH-N-IN) has a mass of $m_{{\rm s},2}=2.221$ g and a reported extension rate of $k_{{\rm s},2}=7$ N/m. The springs are made of music wire and they have cross-over type hooks at their ends. The total mass of the three springs in series is given by $m_{{\rm s}}=8.271$ g. In addition to the extension rates above, the masses of the vertically aligned springs also contribute to the total spring constant of the oscillator. In the case of the higher damping oscillator, where damper fibers are used, there is 3.0 mm loose in the damper fibers so that the oscillator mass is entirely carried by the spring. In our analysis of the experimental results, we use the effective mass, undamped angular frequency and the damping constant as the only parameters of the oscillator. Thus, the total spring constant of the system can be determined from the experimental results as $k=m\omega _0^2$. For the lower damping oscillator, we thus have $k=2.172$ N/m, while for the higher damping oscillator we have $k=2.159$ N/m.

### Michelson interferometer

The motion of the mechanical oscillator is detected by the Michelson interferometer to which the oscillator is connected by setting the oscillator mirror 2 in one of the two interferometer arms. One of the other interferometer arm mirrors is motorized so that it can be used for the remote tuning of the interference fringe spacing. The fringe spacing is adjusted only before the measurements and it is not actively changed during the measurements. The arm length of the interferometer is about 10 cm. All mirrors in the interferometer arms have a reflectivity of over 99% (Thorlabs, BB1-E02). The interferometer utilizes the 5-mW continuous-wave TEM$_{00}$ He–Ne laser (JDSU, 1125P) at 632.8 nm. The laser power is reduced by a factor of 1/10 by a neutral density filter (Thorlabs, NE10A). The dynamics of the interference fringes is recorded by a CMOS camera (Edmund Optics, EO-0413C) with a frame rate of 200 frames per second. The frame size recorded is $600 \times 30$ pixels.

### Tracking the dynamics of interference fringes

The dynamics of the interference fringes is tracked from the recorded video files by observing the movement of the intensity maxima and minima frame by frame. The interference fringes are illustrated in the computer screen of Fig. 1, where the movement of the fringes takes place in the horizontal direction when other disturbances are settled down. If the fringes move a distance that is equal to the distance between two intensity maxima, this indicates that the mechanical oscillator moves half a wavelength in the vertical direction. For efficient analysis of millions of frames, a C++ code was written that utilizes the Open Computer Vision Library (Open CV). The code also detects possible changes in the distance between intensity maxima as these scale changes indicate motion of the oscillator in lateral directions. Since the mechanical oscillator is hanging on a spring, it can move in all three dimensions. However, the interferometer is the most sensitive for the vertical motion of the oscillator that is of our interest. If the oscillator is particularly disturbed, the interference fringes can also rotate and the scale of the fringes can vary. These effects are, however, negligibly small during measurement conditions, when external noise sources are minimized. The frequency responses of these effects are also different from the mechanical resonance frequency of the oscillator so they do not contribute to the magnitude of the observed displacement amplitude.

### Acoustic and seismic isolation

The apparatuses are mounted on an actively damped optical table for isolating the setup from acoustic and seismic vibrations. The mechanical oscillator part of the setup is covered with plastic walls to minimize air flows. The measurements were carried out at nighttime to minimize disturbances in the surroundings of the laboratory.
